# Protective Immunity of the Primary SARS-CoV-2 Infection Reduces Disease Severity Post Re-Infection with Delta Variants in Syrian Hamsters

**DOI:** 10.3390/v14030596

**Published:** 2022-03-13

**Authors:** Sreelekshmy Mohandas, Pragya D. Yadav, Anita Shete, Dimpal Nyayanit, Rajlaxmi Jain, Gajanan Sapkal, Chandrashekhar Mote

**Affiliations:** 1 Indian Council of Medical Research-National Institute of Virology, Pune 411021, India; sreelekshmy88@gmail.com (S.M.); anitaaich2008@gmail.com (A.S.); dimpal.nyayanit@gmail.com (D.N.); rajlaxmijain@gmail.com (R.J.); gajanansapkalniv@gmail.com (G.S.); 2 Department of Veterinary Pathology, Krantisinh Nana Patil College of Veterinary Science, Shirwal 412801, India; drcsmote1@gmail.com

**Keywords:** SARS-CoV-2, Delta AY.1, re-infection, pathogenicity, Syrian hamsters

## Abstract

The Severe Acute Respiratory Syndrome Coronavirus-2 (SARS-CoV-2) Delta variant has evolved to become the dominant SARS-CoV-2 lineage with multiple sub-lineages and there are also reports of re-infections caused by this variant. We studied the disease characteristics induced by the Delta AY.1 variant and compared it with the Delta and B.1 variants in Syrian hamsters. We also assessed the potential of re-infection by these variants in Coronavirus disease 2019 recovered hamsters 3 months after initial infection. The variants produced disease characterized by high viral load in the respiratory tract and interstitial pneumonia. The Delta AY.1 variant produced mild disease in the hamster model and did not show any evidence of neutralization resistance due to the presence of the K417N mutation, as speculated. Re-infection with a high virus dose of the Delta and B.1 variants 3 months after B.1 variant infection resulted in reduced virus shedding, disease severity and increased neutralizing antibody levels in the re-infected hamsters. The reduction in viral load and lung disease after re-infection with the Delta AY.1 variant was not marked. Upper respiratory tract viral RNA loads remained similar after re-infection in all the groups. The present findings show that prior infection could not produce sterilizing immunity but that it can broaden the neutralizing response and reduce disease severity in case of reinfection.

## 1. Introduction

The B.1.617.2 lineage of Severe Acute Respiratory Syndrome Coronavirus-2 (SARS-CoV-2) was first detected in India on 22 September 2020 [1]. The variant was later categorized as a variant of concern (VOC) and was named the Delta variant by the World Health Organization on 31 May 2021 [2]. The variant spread at an alarming rate to become the most dominant SARS-CoV-2 lineage circulating globally and spread to 201 countries by 2 December 2021 [2]. The amino acid substitutions in the spike protein of the Delta variant, such as D614G, T478K, P681R and L452R, are known to affect transmissibility and neutralization [3]. The variant was responsible for the rise in Coronavirus disease 2019 (COVID-19) cases in 2021 in many countries, including India, the United Kingdom, Fiji, South Africa, parts of Asia, the United States, Australia and New Zealand [2]. The Delta variant has been further subdivided into Delta AY.1 and AY.127 according to the Pango lineage designation system. Among these sub-lineages, AY.1 and AY.2 possess the K417N substitution, which is also present in the B.1.351 variant, suggesting that it plays a role in immune evasion. As of 6 December 2021, the AY.1 lineage has been detected in at least 43 countries and AY.2 in 8 countries. AY.1 has amino acid substitutions at T19R, E156G, 157/158 del, W258L, K417N, L452R, T478K, D614G, D950N and P681R [1]. The information on the biological characteristics of these sub-lineages, including transmissibility, disease severity, and immune evasion, are still unknown.

SARS-CoV-2 generates a neutralizing antibody (NAb) response after infection in humans, but the protective immune titre required to prevent subsequent infection is not yet known [4]. In the case of other human coronaviruses (HCoV), waning of immunity is observed in 1 to 3 years and re-infection events have been reported as a common feature of HCoV-NL63, HCoV-229E, HCoV-OC43 and HCoV-HKU1 [5,6]. After natural SARS-CoV-2 human infection, immune response is suspected to persist for about 90 days in most patients [4]. SARS-CoV-2 re-infection cases with varied disease severity have been reported in many countries [7,8,9,10,11]. The speculated reasons for re-infection are infection with a higher virus dose/another virulent strain, antibody-dependent enhancement and waning of immune response [8]. Laboratory studies have shown that the duration of infection-acquired immunity is inconsistent and responses against VOCs also differ [12]. The risk of re-infection also depends on host susceptibility, vaccination status and exposure to COVID-19 patients during the infectious phase [13]. Understanding the potential risk of re-infection is important in improving COVID-19 prevention and control measures. Re-infection studies in the population have not been widely reported and the rate of re-infection is also unclear [14]. There are few reports of aggravated disease severity in the case of Delta variant re-infection [15,16]. The impact of immunity on the threat of re-infection posed by different variants still needs to be understood.

Animal models are important in understanding virus properties, disease pathogenesis, measuring the efficacy of countermeasures, etc. Syrian hamsters have been widely used in studying SARS-CoV-2 disease characteristics. Our previous studies have revealed the pathogenicity and immune-evasive properties of the Delta variant in hamsters [17]. Few animal model studies have demonstrated that the NAb response generated after primary SARS-CoV-2 infection can reduce the viral load and severity of re-infection [18,19]. The degree of protection against re-infection by Delta lineage variants is still unclear. A recent study which evaluated the protection offered by infection-acquired immunity after 15 months in hamsters showed protection against re-infection with the Delta variant and prevention of transmission to naive hamsters [20].

The SARS-CoV-2 B.1 variant possessing the D614G mutation in the spike protein was found to be highly transmissible and became the predominant variant during the early phase of the pandemic [21]. High upper respiratory tract viral load with a lower disease severity has been reported for this variant [21,22]. This ancestral variant with only the D614G mutation in the spike protein has been used as a comparator virus in multiple research studies [22,23]. We have also used the B.1 variant in the present study for the comparison of the disease characteristics of other variants.

Here we have studied the pathogenicity of the Delta AY.1 variant in comparison with the Delta and B.1 variants and also assessed the re-infection potential of these variants in B.1 infection-recovered hamsters 3 months after initial infection. In addition, the neutralization potential of the infected hamster sera was assessed against the B.1, Delta, Delta AY.1, and Beta variants.

## 2. Materials and Methods

### 2.1. Virus and Cells

SARS-CoV-2 variants B.1 (GISAID accession no: EPL_ISL_825084), Delta (GISAID accession no.: EPI_ISL_2400521), Delta AY.1 (GISAID accession no. EPI_ISL_2671901), and Beta (GISAID accession no. EPI_ISL_2036294) isolated from nasopharyngeal swabs of COVID-19 patients were used for the study. The isolates were passaged twice in Vero (ATCC^®^ CCL-81^TM^) cells (ATCC, Manassas, VA, USA) and titrated to measure the 50% tissue culture infective dose (TCID50) as per the Reed and Muench method. The variants used in the study had the following amino acid substitutions in the spike protein. Delta AY.1 variant had D614G, E156G, F157del, K417N, L452R, P681R, R158del, T19R, T95I and T478K substitutions; the Delta variant had A222V, D614G, D950N, G142D, L452R, P681R, T19R, T478K substitutions; and the B.1 variant had the D614G substitution in the spike protein.

### 2.2. Animal Experiments

**Figure 1:**
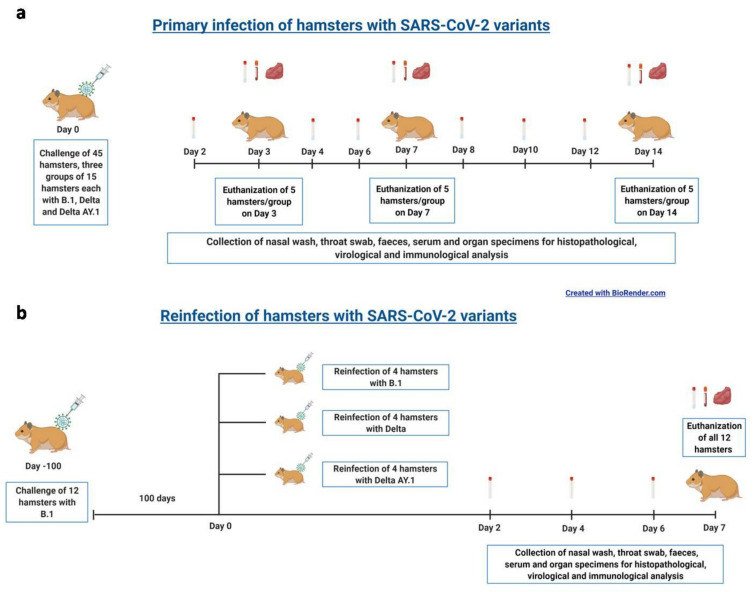
Study design. (**a**) Summary of the Delta AY.1 vs. Delta and B.1 pathogenicity study. (**b**) Summary of the reinfection process.

The experiments were performed in the Containment Facility of ICMR-National Institute of Virology, Pune. Three study groups of 17 female Syrian hamsters (procured from a CPCSEA-authorized breeding facility) 12–14 weeks old were included in the study to assess pathogenicity/virus shedding. A virus dose of 10^5^ TCID50 (0.1 mL volume) of the Delta/Delta AY.1/B.1 variants was used intranasally to inoculate the hamsters (Figure 1a). Throat swab, nasal wash and faeces samples (*n* = 7) were collected on alternate days during the study period. Hamsters were observed for a period of 14 days for body weight loss, and five hamsters/group were sacrificed 3, 7 and 14 days post infection (DPI) to collect organs (lungs, nasal turbinates, heart, liver, kidney, intestine, spleen and brain) and blood samples.

For the re-infection study, 12 female hamsters, 16–18 weeks old, that were previously infected with the B.1 variant of SARS-CoV-2 (with an infectious dose of 10^4.5^ TCID50) were used 3 months after initial infection (Figure 1b). IgG response and NAb levels were assessed and the animals were randomly divided into three groups (four animals per group). The hamsters were re-infected with the Delta/Delta AY.1/B.1 variants with a virus dose of 10^5^ TCID50 (0.1 mL volume intranasally). Throat swab, nasal wash and faeces samples were collected on 2, 4, 6 DPI and body weight change was monitored for 7 days. The hamsters were sacrificed on 7 DPI to collect lungs, nasal turbinates and blood samples.

### 2.3. Viral Load Estimation

Nasal wash, throat swab and organ tissue samples were used for viral load estimation. Organ samples collected during necropsy were weighed and homogenized in sterile media using beads in a tissue lyser machine (Qiagen, Hilden, Germany). The lysate was used for RNA extraction using the MagMAX™ Viral/Pathogen Nucleic Acid Isolation Kit (Thermo Fisher Scientific, Waltham, MA, USA) as per the manufacturer’s instructions. Quantitative real-time RT-PCR was performed for the E gene of SARS-CoV-2 using published primers to estimate the genomic viral RNA (gRNA) load and for the N gene of SARS-CoV-2 using published primers to estimate the subgenomic viral RNA (sgRNA) load [24,25]. The lung samples collected 7 days post primary infection and re-infection were used for virus titration in Vero (ATCC^®^ CCL-81^TM^) cells (ATCC, Manassas, VA, USA). Lung tissue homogenates were centrifuged at 1984× *g* for 10 min and 0.1 mL of the supernatant was used for the titration. The supernatant was added onto 24-well tissue culture plate cell monolayers and incubated at 37 °C. The cells were washed with phosphate-buffered saline after the incubation period of one hour. Maintenance media containing 2% fetal bovine serum (Sigma Aldrich, St. Louis, MO, USA) was added onto the cells and further incubated in a CO_2_ incubator at 37 °C. The cells were examined for cytopathic effects for 4 days. The titres were determined by the Reed and Muench method.

### 2.4. Anti-SARS-CoV-2 IgG Detection

The serum samples were tested for IgG antibodies by an in-house developed qualitative ELISA [26]. Briefly, inactivated SARS-CoV-2 antigen/Vero (ATCC^®^ CCL-81^TM^) cell lysate-coated microtitre plates were blocked with liquid plate sealer. The diluted hamster sera samples (1:100 to 1:10,000) were added and incubated for 60 min at 37 °C. The plates were washed following incubation and 1:3000 dilution of anti-hamster IgG horseradish peroxidase (Thermo Fisher Scientific, Waltham, MA, USA) was added and incubated for 60 min. The plates were washed and substrate was added to each well for color development. The reaction was terminated with sulfuric acid and the absorbance was measured at 450 nm using an ELISA reader. The assay was performed in duplicate and the assay cutoff was set at an optical density (OD) value of 0.2 and positive/negative ratio of 1.5.

### 2.5. Serum Neutralizing Antibody Level Estimation

A plaque reduction neutralization test (PRNT) was performed against the B.1, Delta, Delta AY.1 and Beta variants, as described previously [27]. Diluted sera were mixed with 50–60 plaque forming units/0.1 mL virus and the virus–sera mixture was incubated for 60 min. The mixture was then added on to a tissue culture plate with a Vero (ATCC^®^ CCL-81^TM^) monolayer (ATCC, Manassas, VA, USA). After 60 min, the mixture was aspirated and media with 2% carboxymethyl cellulose with 2% fetal bovine serum (Sigma Aldrich, St. Louis, MO, USA) was added. After an incubation period of 4 days, the media was decanted and amido black staining was performed. The plaques were counted and PRNT50 titres were calculated.

### 2.6. Serum Cytokine Level Estimation

ELISA-based estimation (Immunotag, St. Louis, MO, USA) was performed to assess the levels of IL-4, IL-6, IL-10, IFN-γ and TNF-α in hamster sera samples as per the manufacturer’s instructions.

### 2.7. Lung Histopathological Evaluation

Formalin-fixed lung tissue samples were processed using an automated tissue processor and were stained by routine hematoxylin and eosin staining. The samples were coded and blindly scored by a pathologist. The bronchiolar (degeneration, epithelial loss), alveolar parenchymal (edema, exudation, mononuclear infiltration, emphysema, pneumocyte hyperplasia, septal thickening) and vascular lesions (congestion, hemorrhages, perivascular infiltrations) were graded for severity on a score from 0 to 4 (0 = no changes, 1 = minimal, 2 = mild, 3 = moderate, 4 = severe). The cumulative scores for each group on 3, 7 and 14 DPI were compared and statistically analyzed. The scores of the re-infected animals on 7 DPI were compared with the scores of the primary infection group on 7 DPI.

### 2.8. Data Analysis

GraphPad Prism version 9.2.0 (GraphPad software Inc., San Diego, CA, USA) software was used for the descriptive statistics and statistical analysis. Nonparametric Mann–Whitney tests were used for the analysis. *p*-values less than 0.05 were considered statistically significant. For the primary infection study, the comparison was performed daywise among Delta, Delta AY.1, and B.1 infected groups for viral load, virus shedding, body weight loss, NAb titres and histopathological scores. For cytokine response, the comparison was performed with the uninfected control hamster sera. For the re-infection study with each variant, the comparison was performed with the data for the primary infection of the corresponding variant.

## 3. Results

### 3.1. Body Weight Changes in Hamsters after Primary Infection

**Figure 2:**
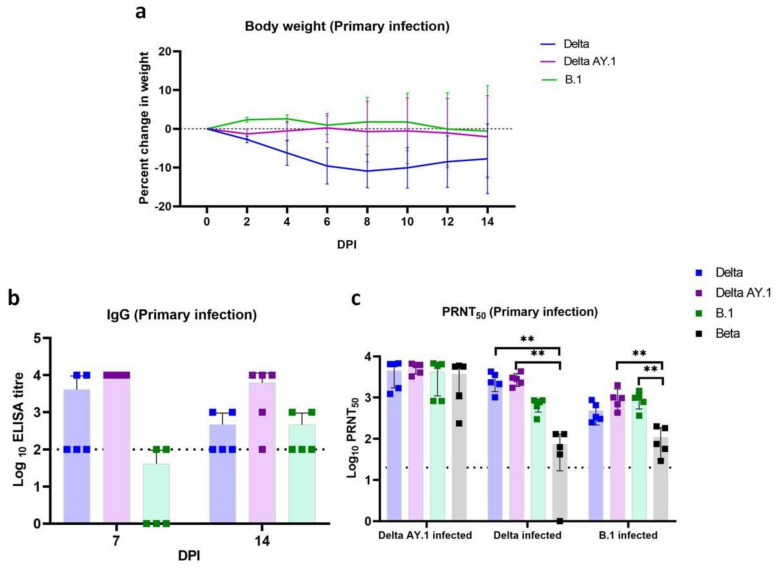
Body weight loss and immune response in hamsters post infection. (**a**) Line graph depicting body weight loss in hamsters post first infection. (Delta vs. Delta AY.1 on 6 DPI, *p* = 0.0248 and Delta vs. B.1 on 6 DPI, *p* = 0.0082). The error bars depict the standard deviation (SD). (**b**) Scatter plot depicting IgG ELISA titre in hamsters post first infection (*n* = 5) on days 7 and 14. Scatter plot depicting (**c**) PRNT50 titres against variants in Delta AY.1-infected hamsters (*n* = 5), Delta-infected hamsters, (*p* = 0.0066 (Delta vs. Beta), Mann–Whitney test, *n* = 5) and B.1-infected hamsters (*p* = 0.0080 B.1 vs. Beta, Mann–Whitney test, *n* = 5). The bars represent the mean and the error bars depict the SD. Limit of detection of assay is depicted as the dotted line and ** represents *p* value < 0.001.

The Delta AY.1- and B.1-infected group animals showed the maximum weight loss [mean ± standard deviation (SD)] of −2.048 ± 8.557% and −0.566 ± 9.432%, respectively, on 14 DPI, whereas in the Delta variant-infected group, the peak weight loss (−10.886 ± 3.46%) was observed on 8 DPI. The body weight loss observed in the Delta AY.1 group was significantly lesser in comparison to the Delta variant infection (Figure 2a).

### 3.2. Immune Response in Hamsters after Primary Infection

Anti-SARS-CoV-2 IgG response could be observed from day 7 in all primary infected groups (Figure 2b). A mean optical density (OD) ± SD of 0.65 ± 0.45, 1.00 ± 0.67, 1.02 ± 0.40 on 7 DPI and 1.25 ± 0.55, 0.62 ± 0.13 and 0.62 ± 0.11 on 14 DPI for the Delta AY.1, Delta and B.1 variant, respectively, was observed with 1:100 dilution of sera. Cross NAbs were detected in hamster sera against the Delta AY.1, Delta, B.1 and Beta variants (Figure 2c). The mean ± SD of NAb titre on 14 DPI in the Delta AY.1-infected group against the Delta AY.1, Delta, B.1 and Beta variants were 5265 ± 1504, 4544 ± 2824, 4159 ± 3062 and 3735 ± 2804, respectively. In the case of the Delta- and B.1-infected groups, mean NAb responses against the Delta AY.1, Delta, B.1 and Beta variants were 2848 ± 978, 2667 ± 1275, 680 ± 234, 73 ± 57 and 1020 ± 59, 485 ± 269, 925 ± 398 and 109 ± 77, respectively. Thus, a significant reduction in NAb titre against the Beta variant was observed with the Delta and B.1 variant infected animal sera, whereas a comparable response was observed in the case of the AY.1-infected group.

The serum IL-6 level was elevated in all the infected groups. The B.1 group hamsters showed a significantly higher IL-6 level on 3, 7 and 14 DPI than the Delta group on 14 DPI (Supplementary Figure S1). In the case of the Delta AY.1 group, the increase was not statistically significant. Among the other cytokines analyzed in serum, i.e., IL-4, IL-10, IFN-γ and TNF-α, no significant increase was observed in comparison to the control animal sera.

### 3.3. Viral Shedding in Hamsters after Primary Infection

**Figure 3:**
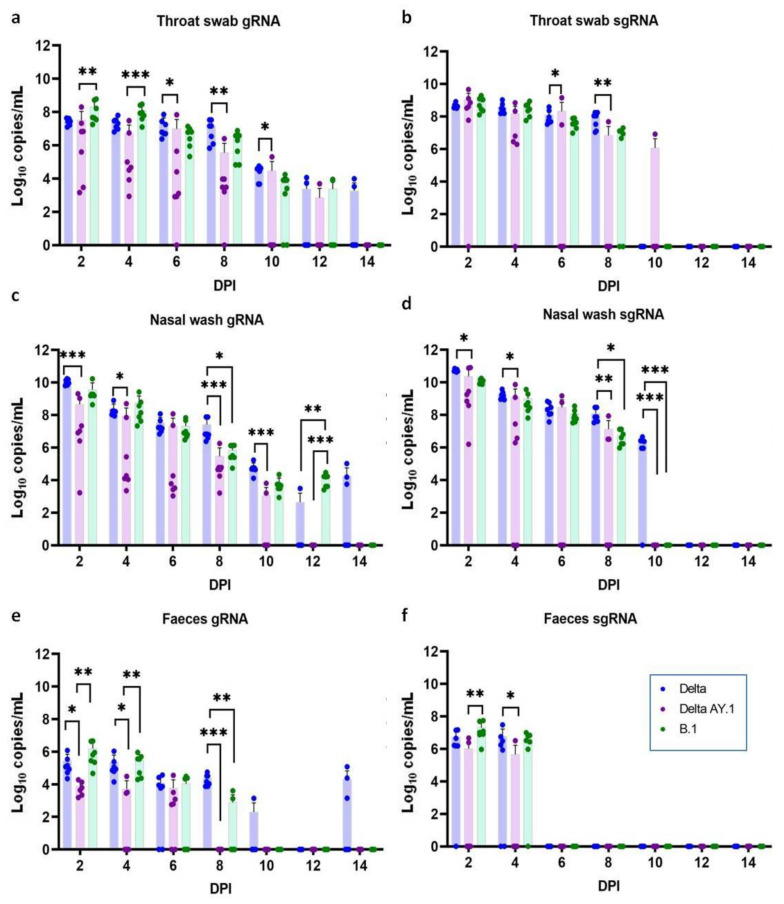
SARS-CoV-2 viral RNA shedding in hamsters after infection. Scatter plot depicting viral gRNA load in the (**a**) throat swabs (**c**) nasal washes and (**e**) faeces of hamsters post infection, (Mann–Whitney test, *n* = 7). Scatter plot depicting viral sgRNA load in (**b**) throat swabs, (**d**) nasal washes and (**f**) faeces of hamsters post infection, (Mann–Whitney test, *n* = 7). The bars represent the mean and the error bars depict the standard deviation. The *p* values < 0.05, <0.001 and <0.0001 are represented as *, ** and *** respectively.

In the throat swab and the nasal wash samples, genomic RNA (gRNA) was detected with a decreasing trend until 12 DPI in the Delta AY.1- and the B.1-infected groups and until 14 DPI in the Delta variant-infected group. The average viral gRNA levels in the throat swab were significantly lower in the Delta AY.1-infected group on 6, 8 and 10 DPI, as were the subgenomic (sg) RNA levels on 8 DPI in comparison with the Delta variant group (Figure 3a,b). The nasal wash and faeces viral RNA loads were also significantly lower in the Delta AY.1-infected group (Figure 3c–f).

### 3.4. Viral Load in Organs in Hamsters after Primary Infection

In the nasal turbinates, gRNA and sgRNA could be detected until 14 DPI in all the groups. The Delta AY.1 group did not show any significant differences in the gRNA or sgRNA levels of the nasal turbinates in comparison with the other groups (Supplementary Figure S2a,b). gRNA and sgRNA was detected in the lungs of hamsters in both the Delta and B.1 groups until 14 DPI. In the Delta AY.1 group, a comparatively lower viral load (mean ± SD = 4.55 × 10^4^ ± 9.0 × 10^4^) was observed on 7 DPI in the lungs, and complete clearance was seen by 14 DPI (Supplementary Figure S2c,d). Other organs, such as the brain (1/5), heart (2/5) and large intestine (2/5), in hamsters in the Delta AY.1-infected group showed sgRNA positivity on 3 DPI but none of the hamsters in the Delta- and B.1-infected groups showed positivity in non-respiratory organs.

### 3.5. Lung Pathological Changes after Primary Infection

Grossly, the lungs of 2/10 animals in the Delta AY.1 group and of 7/10 animals in the Delta variant group sacrificed on 7 and 14 DPI after the primary infection showed hemorrhagic lesions. A few focal hemorrhages were only seen in the case of B.1 infection. On 3 DPI, the vascular pathological changes in the lungs were minimal in all groups, and mild pneumonic changes were observed in the alveolar parenchyma. The pneumonic changes were minimal in the Delta AY.1 group on 7 DPI, which became pronounced by 14 DPI (in three out of five animals infected). By 7 DPI, inflammatory changes became severe in the Delta variant group, characterized by severe congestion/hemorrhages, alveolar consolidation, loss of bronchial epithelium, septal thickening, pneumocyte hyperplasia and cellular infiltration in the alveolar interstitial space, peribronchial and perivascular area. In the case of B.1-infected hamsters, pneumonic changes became more pronounced by 7 DPI. The highest lung cumulative score was observed in the Delta-infected group on 7 DPI (Supplementary Figure S2e).

### 3.6. Reduced Disease Severity in Hamsters Post Re-Infection

**Figure 4:**
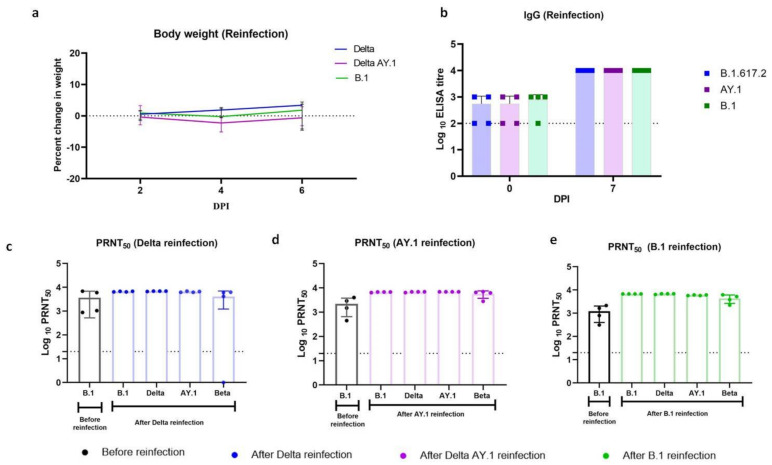
Body weight loss and immune response in hamsters post re-infection. (**a**) Line graph representing percent body weight change in hamsters on 2, 4 and 6 DPI. The error bars depict the standard deviation (SD). (**b**) Scatter plot depicting IgG ELISA titre in hamsters before and after re-infection on days 0 and 7. PRNT50 titres in (**c**) Delta-, (**d**) Delta AY.1- and (**e**) B.1-infected hamsters before and after re-infection on 7 DPI. The bars represent the means and the error bars depict the SDs. Limit of detection of the assay is shown by the dotted line.

After re-infection, body weight loss in all the infected groups was minimal irrespective of the variant used for infection (Figure 4a). The mean (±SD) body weight change on 7 DPI post re-infection was 1.74 (±3.9), 0.67 (±3.8) and −0.3 (±3.05)% in the Delta, Delta AY.1 and B.1 groups, respectively. The geometric mean IgG titre as determined by ELISA was 316, 316 and 562.3 against the Delta, Delta AY.1 and B.1 variants, respectively, on the day of re-infection, and rose to 10^4^ on 7 DPI in all the groups (Figure 4b). Post re-infection, the PRNT50 titres showed a rise in titres against all the variants, i.e., B.1, Beta, Delta and Delta AY.1 variants (Figure 4c–e). Serum IL-6 levels did not show any significant elevation, as in the case of the primary infection in the re-infected groups. Other cytokines did not show any significant increase either (Supplementary Figure S3).

In the re-infected animals, only focal hemorrhagic foci were seen in the lungs, contrary to the pronounced gross lesions seen with primary infection (Supplementary Figure S4). The lung–body weight ratio, which was found to have increased in the Delta variant hamsters after primary infection, was found not to have increased in the re-infected hamsters (Supplementary Figure S5).

**Figure 5:**
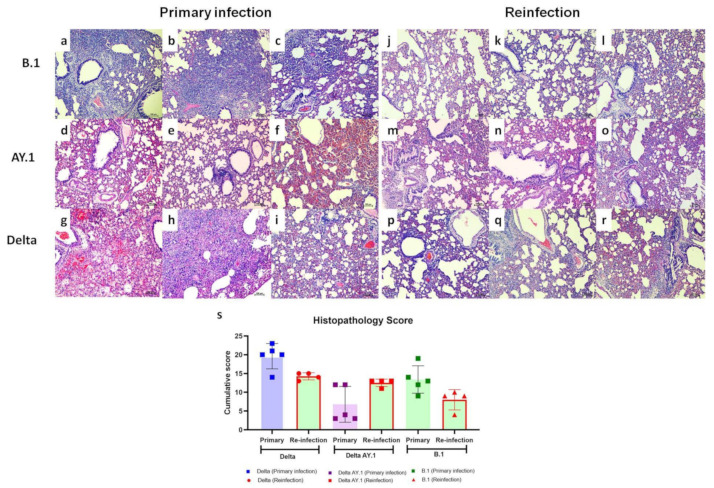
Histopathological changes observed in lungs after primary infection and re-infection. Lung sections from hamsters infected with the B.1 variant showing (**a**,**b**) Diffuse interstitial pneumonia and (**c**) large foci of alveolar interstitial thickening, inflammatory cell infiltration, loss of bronchial epithelium and congestion in the parenchyma on 7 DPI after primary infection, as revealed by H&E staining, scale bar = 100 µm. Lung sections from hamsters infected with the Delta AY.1 variant showing (**d**) diffuse alveolar capillary engorgement, (**e**) peribronchial inflammatory cell infiltration and (**f**) diffuse alveolar capillary engorgement and hemorrhages on 7 DPI after primary infection, H&E, scale bar = 100 µm. Lung sections from hamsters infected with the Delta variant showing (**g**) diffuse alveolar damage with hemorrhage in the parenchyma, (**h**) diffuse alveolar septal thickening and exudation and (**i**) alveolar interstitial thickening, congestion and peribronchial inflammatory cell infiltration on 7 DPI after primary infection, H&E, scale bar = 100 µm. Lung sections from hamsters re-infected with the B.1 variant showing (**j**,**k**) a small amount of focal alveolar interstitial thickening and (**l**) alveolar interstitial thickening and congestion on 7 DPI after re-infection, H&E, scale bar = 100 µm. Lung sections from hamsters re-infected with the Delta AY.1 variant showing (**m**–**o**) diffuse alveolar interstitial thickening, congestion and inflammatory cell infiltration on 7 DPI after re-infection, H&E, scale bar = 100 µm. Lung sections from hamsters infected with the Delta variant showing (**p**) diffuse congestive changes, (**q**) alveolar septal thickening, peribronchial inflammatory cell infiltration and loss of bronchial epithelium and (**r**) diffuse engorgement, hemorrhages and peribronchial inflammatory cell filtration on 7 DPI post re-infection, H& E, scale bar = 100 µm. (**s**) Scatter plot depicting cumulative lung histopathology scores in hamsters post primary infection and re-infection with Delta, Delta AY.1 and B.1 on 7 DPI. The samples were scored on a scale of 0 to +4 for vascular changes, bronchial changes, alveolar parenchymal changes and inflammatory cellular infiltration. The bars represent the means and the error bars the standard deviations.

In the case of the Delta- and B.1-re-infected group, the lung pathological changes observed on 7 DPI were milder in comparison to those observed with primary infection. The lungs from the B.1-re-infected group showed mild alveolar parenchymal inflammatory cell infiltration, septal thickening and bronchiolar epithelial loss, whereas the lungs from the hamsters of the Delta variant-re-infected group showed mild bronchial/vascular changes and moderate alveolar changes (consolidation, alveolar septal thickening and pneumocyte hyperplasia). In the case of AY.1-re-infected animals, similar disease severity to that of two out of five naive AY.1-infected animals were observed, characterized by mild congestion/hemorrhages and moderate alveolar parenchymal consolidation, septal thickening and pneumocyte hyperplasia, as well as mononuclear cellular infiltration (Figure 5).

### 3.7. Reduced Viral RNA Shedding and Lung Viral RNA Load in Re-Infected Hamsters

**Figure 6:**
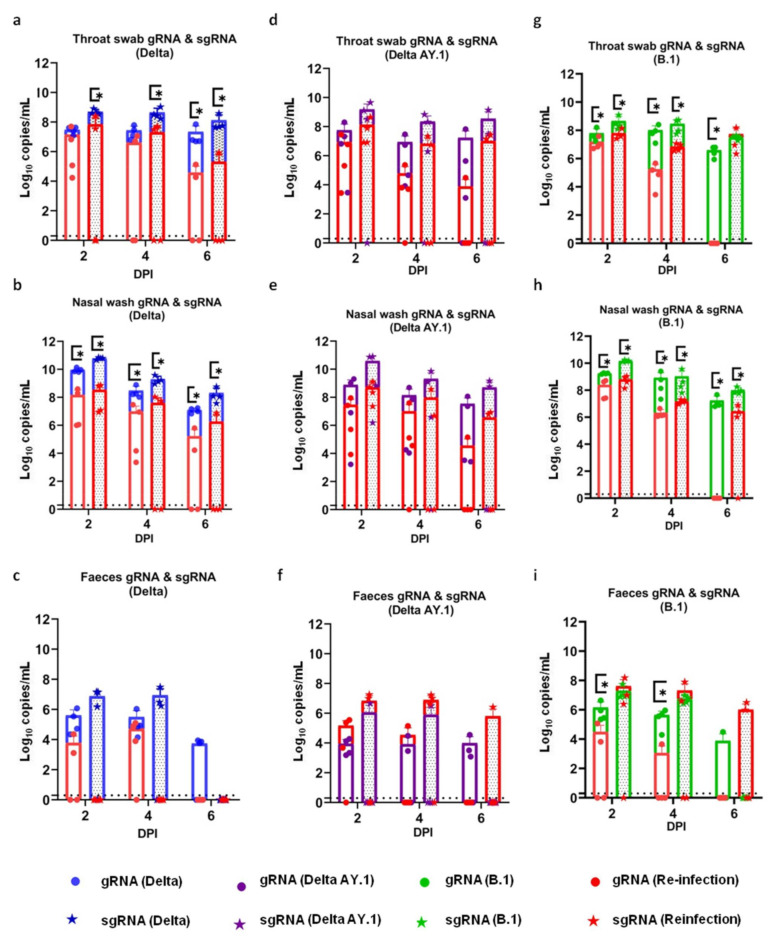
SARS-CoV-2 viral RNA shedding in hamsters after re-infection. Superimposed scatter plot depicting viral gRNA and sgRNA load in (**a**) throat swabs (*p* = 0.0286, Mann–Whitney test, *n* = 4) and (**b**) nasal washes (*p* = 0.0286, Mann–Whitney test, *n* = 4) and (**c**) faeces of the Delta variant group; the (**d**) throat swabs, (**e**) nasal washes and (**f**) faeces of the Delta AY.1 group; and (**g**) the throat swabs (*p* = 0.0286, Mann–Whitney test, *n*=4), (**h**) nasal washes (*p* = 0.0286, Mann–Whitney test, *n* = 4) and (**i**) faeces (*p* = 0.0286, Mann–Whitney test, *n* = 4) of the B.1-infected hamsters during primary infection and re-infection on 7 DPI. The bars represent the means and the error bars the standard deviations. *p* value < 0.05 is represented as *.

The viral RNA shedding through the nasal and oral cavity was reduced post re-infection in the hamsters. On 2, 4 and 6 days post re-infection, viral RNA shedding in the throat swabs and nasal washes was significantly lesser in the Delta- and B.1-re-infected hamsters in comparison to the viral RNA load observed in hamsters post primary infection (Figure 6a–f). In the faeces samples of the B.1- and Delta-re-infected groups, also, the reduction was evident. Although the viral RNA load in the AY.1 group showed a reduction, the values were not statistically significant.

**Figure 7:**
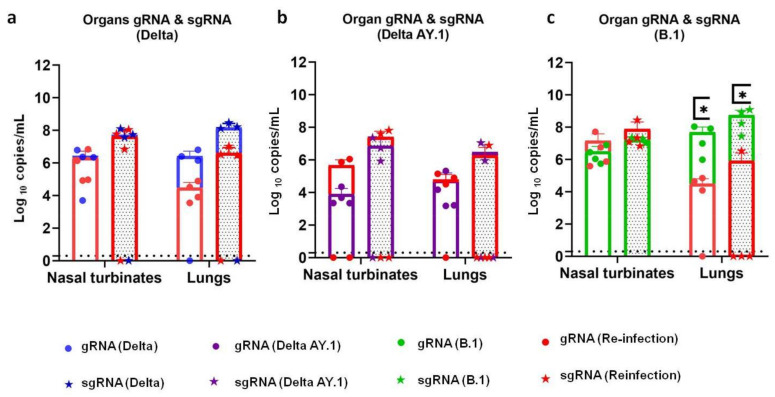
SARS-CoV-2 viral RNA load in organs after re-infection. Superimposed scatter plot depicting viral gRNA and sgRNA loads in the nasal turbinates and lungs of (**a**) Delta-, (**b**) Delta AY.1- and (**c**) B.1- (*p* = 0.0286, Mann–Whitney test, *n* = 4) infected hamsters during primary infection and re-infection on 7 DPI. The bars represent the means and the error bars the standard deviations. *p* value < 0.05 is represented as *.

Unlike the viral RNA shed through the nasal cavity, the viral RNA load in the nasal turbinates was comparable in both the re-infected and the primary infected groups on 7 DPI in all the groups (Figure 7a–c). However, the viral RNA load in the lungs was reduced. Lung gRNA levels were significantly lower in the B.1-re-infected group, whereas in the case of Delta variant re-infection, only a minimal reduction was seen, and in the AY.1 group a viral RNA load was observed comparable to that of the primary infection on 7 DPI. Live virus titration was performed on the lung samples collected on 7 DPI from all the groups and no titre could be detected.

## 4. Discussion

The Delta variant and its sub-lineages have become the dominant SARS-CoV-2 lineages world-wide. The higher rate of transmissibility, increased disease severity and immune evasion potential of the Delta variant has alerted the scientific community to be vigilant about the mutating variants [2,3]. We have studied properties of the Delta AY.1 variant in comparison with the Delta and B.1 variants in Syrian hamsters and the potential for re-infection in hamsters by Delta variants 3 months post recovery from SARS-CoV-2 infection, the period for which antibodies are reported to persist in humans after infection.

Delta AY.1 variant infection in Syrian hamsters produced mild disease characterized by negligible weight loss, lower viral load in upper and lower respiratory tracts and mild pneumonic changes in the lungs. Comparable cell entry efficiency was reported by a recent study for the Delta sub-lineages with K417N mutation in comparison with the wildtype SARS-CoV-2 with the D614G mutation. This mutation tends to affect ACE2 binding affinity moderately [28]. The lower prevalence rate of the variant worldwide, i.e., less than 0.5% to date after its initial detection, points to the less efficient transmission/binding affinity of the variant [3]. Increased disease severity or risk of hospitalization has been reported for Delta variant infection compared to the earlier SARS-CoV-2 strains [29,30]. We also observed a higher degree of disease severity induced by the Delta variant in hamsters. Pathological changes observed in the lungs were similar to those reported during the acute phase of infection in humans [31,32]. Diffuse congestion and hemorrhages were observed grossly in the lungs and the histopathological changes were mostly of epithelial and vascular types. Microthrombi formation was not observed in the lungs of hamsters, unlike humans. Hamsters exhibited weight loss as the characteristic symptom of severe disease, unlike flu like symptoms and respiratory distress in humans [31].

The K417N mutation in the Delta AY.1 variant was found to be critical for neutralization resistance against some potent NAbs against SARS-CoV-2 [33,34]. Here, we observed a comparable neutralization efficiency in Delta AY.1-infected hamster sera against the Delta, B.1 and Beta variants, suggesting that the presence of the K417N mutation may not confer an advantage in terms of immune evasion, at least against these variants. Similar results of comparable neutralization efficiency of the Delta variant with the K417N mutation and the Delta variant has been reported in pseudo-virus neutralization studies [28]. Yadav et al. 2021 have reported comparable neutralization for the Delta AY.1 and Delta variants with the sera of naive BBV152 vaccinees, recovered cases with full vaccination and breakthrough cases in comparison to the B.1 variant [35]. The available vaccines against SARS-CoV-2 have shown reduced protection against symptomatic disease/infection by the Delta variant [2]. We observed cross NAbs against the variants studied here after infection and a boost in titres after re-infection. However, a significantly lower neutralization titre was observed against the Beta variant in the case of the Delta and B.1 variant-infected animal sera after primary infection. The B.1.351 variant is known worldwide for its immune escape property due to the mutations K417N, E484K and N501Y in the RBD of the spike region [33].

Many cytokines have been reported to be increased in severe COVID-19 patients and a few of them, such as IL-6, IL-8, IL-10 and TNF-α, are considered to be indicators of severe disease [36,37]. The increased production of cytokines can lead to cytokine storm and worsening of the disease prognosis [38]. IL-6, IL1-beta and TNF-α increase has been reported in hamsters infected with SARS-CoV-2 [39]. Here, we have observed increased IL-6 cytokine levels after primary infection in hamsters with SARS-CoV-2 variants. IL-6 is an important cytokine in host responses against viral infection [40]. The increase in IL-6 levels observed here could have contributed to the host response in control of infection. Other inflammatory cytokines, such as IL-4, IFN-γ and TNF-α, were also increased in comparison to control animals during the acute phase of infection. IL-6 level increase was independent of the lung histopathological score. The B.1 variant-infected animals showed the highest average lung sgRNA loads on 7 and 14 DPI and the highest increase in serum IL-6 levels. We found no aggravation in cytokine responses post re-infection. A detailed study of cell-mediated response was not possible because of the non-availability of reagents specific for hamsters.

The protective immunity conferred by prior SARS-CoV-2 infection is similar to that of vaccination [41]. Natural infection generates an effective mucosal immune response, unlike intramuscular vaccination [42]. Re-infection with SARS-CoV-2 and other human coronaviruses has been reported [6,7,8,9,10,11]. The re-infections are common 12 months after initial infection in the case of seasonal HCoVs. A trend of reduced virus replication and an increase in NAb titres were observed in hamsters following re-infection irrespective of the variants used for infection. The neutralizing titres against the Beta variant were also comparable following re-infection, contrary to the differences observed after primary infection. Polyclonal antibody response is generated by natural infection. Antibody evolution through somatic mutations and repeated antigen exposure could improve protection against newer emerging variants [43]. Thus, reinfections tend to improve neutralizing abilities against multiple variants.

Earlier research has shown that prior COVID-19 infection reduces virus replication and thus decreases transmission efficiency in Syrian hamsters re-infected 29 days after initial infection [19]. A recent re-infection study performed 15 months post primary infection in hamsters demonstrated protection against lung disease caused by the Delta variant and the prevention of transmission to naive hamsters [20]. Even though a reduction was seen in viral shedding, the nasal turbinate viral load remained comparable in the present study. We have used a high virus dose of 10^5^ TCID50 for reinfection studies here which also might have contributed to this. This finding highlights the importance of maintaining COVID-19-appropriate behavior till herd immunity is achieved in the population. Reinfection studies with lower virus doses and studies on the persistence of mucosal responses after infection need to be explored for better understanding of respiratory tract protection following re-infection. The hypothesis of high virus dose infection as a cause of re-infection is proved here. Furthermore, studies with lower virus doses for primary infection should be explored to understand whether the protective immune response generated by a low virus dose exposure could prevent further infection, as the majority of human SARS-CoV-2 infections are mild and asymptomatic.

Prior infection did not confer sterilizing immunity in the present study, as reported earlier in rhesus macaques and hamster models [18,43,44]. These studies were performed within a month after recovery from the primary infection, in contrast to our study. Experimentally re-infected or vaccinated animals can shed SARS-CoV-2 through the upper respiratory tract [19,44,45,46,47]. The exact immune correlates of protection from infection are still not known. Other than the NAb levels post infection, the cellular as well as the local mucosal response tends to play an important role in protection against reinfection [48]. Limited mucosal immunity could be a probable reason for the high viral RNA load observed in the upper respiratory tract.

There are reports of varying disease severity in re-infected individuals [9,11,15,16]. Wang et al., 2021 have reported 68.8%, 18.8% and 12.5% of similar, worse and mild disease severity in re-infection cases [11]. Severe disease has also been reported with Delta variant re-infection [15,16]. We did not observe any aggravation of lung disease in the re-infected animals with any of the variants used in this study. In the case of the Delta AY.1-re-infected group, sgRNA clearance was observed in 75% of the animals by 7 DPI but the average viral RNA load remained comparable to that of primary infection and with the viral RNA load of animals of the Delta- as well as the B.1-re-infected group. The lung histopathology score severity was similar to that of the Delta variant-re-infected group in the animals. Considering all these observations, it seems that there was some amount of protection conferred, and a study with a large sample size would be appropriate to reach more definitive conclusions.

To conclude, the Delta AY.1 variant produced mild disease in a hamster model and did not show any evidence of neutralization resistance, as speculated previously. Re-infection with higher virus doses of the Delta variant or the B.1 variant 3 months after B.1 variant infection reduced virus shedding/disease severity and increased NAb levels, irrespective of the variants studied. The findings of this study indicate that primary SARS-CoV-2 infection can reduce the severity of secondary infection by the Delta variant, although it cannot confer sterilizing immunity or guarantee protection from a secondary infection.

## Data Availability

All the data related to the study are available in the manuscript.

## Supplementary Materials

The following supporting information can be downloaded at: https://www.mdpi.com/article/10.3390/v14030596/s1, Figure S1: Serum cytokine levels in hamsters post SARS CoV-2 infection; Figure S2: SARS-CoV-2 viral RNA load in organs of hamster’s post infection; Figure S3: Serum cytokine levels in hamsters post SARS CoV-2 re-infection; Figure S4: Pathological changes observed in lungs after primary infection and re-infection; Figure S5: Lungs body weight ratio in hamsters after primary infection and re-infection.

## References

[1] (2021). PANGO Lineages. https://cov-lineages.org/global_report_B.1.617.2.html.

[2] World Health Organization (2021). https://www.who.int/publications/m/item/weekly-epidemiological-update-on-covid-19---16-november-2021.

[3] Pan American Health Organisation (2021). https://www.paho.org/en/documents/epidemiological-update-coronavirus-disease-covid-19-2-december-2021.

[4] Ju B., Zhang Q., Ge J., Wang R., Sun J., Ge X., Yu J., Shan S., Zhou B., Song S. (2020). Human neutralizing antibodies elicited by SARS-CoV-2 infection. Nature.

[5] Woo P.C., Lau S.K., Wong B.H., Chan K.H., Chu C.M., Tsoi H.W., Huang Y., Peiris J.M., Yuen K.Y. (2004). Longitudinal profile of immunoglobulin G (IgG), IgM, and IgA antibodies against the severe acute respiratory syndrome (SARS) coronavirus nucleocapsid protein in patients with pneumonia due to the SARS coronavirus. Clin. Vaccine Immunol..

[6] Edridge A.W., Kaczorowska J., Hoste A.C., Bakker M., Klein M., Loens K., Jebbink M.F., Matser A., Kinsella C.M., Rueda P. (2020). Seasonal coronavirus protective immunity is short-lasting. Nat. Med..

[7] Prado-Vivar B., Becerra-Wong M., Guadalupe J.J., Marquez S., Gutierrez B., Rojas-Silva P., Grunauer M., Trueba G., Barragan V., Cardenas P. COVID-19 Re-Infection by a Phylogenetically Distinct SARS-CoV-2 Variant, First Confirmed Event in South America. https://ssrn.com/abstract=3686174.

[8] Tillett R.L., Sevinsky J.R., Hartley P.D., Kerwin H., Crawford N., Gorzalski A., Laverdure C., Verma S.C., Rossetto C.C., Jackson D. (2021). Genomic evidence for reinfection with SARS-CoV-2: A case study. Lancet Infect. Dis..

[9] Van Elslande J., Vermeersch P., Vandervoort K., Wawina-Bokalanga T., Vanmechelen B., Wollants E., Laenen L., André E., Van Ranst M., Lagrou K. (2021). Symptomatic severe acute respiratory syndrome coronavirus 2 (SARS-CoV-2) reinfection by a phylogenetically distinct strain. Clin. Infect. Dis..

[10] To K.K., Hung I.F., Ip J.D., Chu A.W., Chan W.M., Tam A.R., Fong C.H., Yuan S., Tsoi H.W., Ng A.C. (2020). COVID-19 re-infection by a phylogenetically distinct SARS-coronavirus-2 strain confirmed by whole genome sequencing. Clin. Infect. Dis..

[11] Wang J., Kaperak C., Sato T., Sakuraba A. (2021). COVID-19 reinfection: A rapid systematic review of case reports and case series. J. Investig. Med..

[12] Wang P., Casner R.G., Nair M.S., Wang M., Yu J., Cerutti G., Liu L., Kwong P.D., Huang Y., Shapiro L. (2021). Increased resistance of SARS-CoV-2 variant P. 1 to antibody neutralization. Cell Host Microb..

[13] Centre for Disease Control and Prevention (2021). https://www.cdc.gov/coronavirus/2019-ncov/hcp/duration-isolation.html.

[14] Vitale J., Mumoli N., Clerici P., De Paschale M., Evangelista I., Cei M., Mazzone A. (2021). Assessment of SARS-CoV-2 Reinfection 1 Year After Primary Infection in a Population in Lombardy, Italy. JAMA Intern. Med..

[15] Shastri J., Parikh S., Aggarwal V., Agrawal S., Chatterjee N., Shah R., Devi P., Mehta P., Pandey R. (2021). Severe SARS-CoV-2 Breakthrough Reinfection with Delta Variant After Recovery from Breakthrough Infection by Alpha Variant in a Fully Vaccinated Health Worker. Front. Med..

[16] Shastri J., Parikh S., Agrawal S., Chatterjee N., Pathak M., Chaudhary S., Sharma C., Kanakan A., Srinivasa Vasudevan J., Maurya R. (2021). Clinical, Serological, Whole Genome Sequence Analyses to Confirm SARS-CoV-2 Reinfection in Patients from Mumbai, India. Front. Med..

[17] Mohandas S., Yadav P.D., Shete A., Nyayanit D., Sapkal G., Lole K., Gupta N. (2021). SARS-CoV-2 Delta variant pathogenesis and host response in Syrian hamsters. Viruses.

[18] Brustolin M., Rodon J., Rodríguez de la Concepción M.L., Ávila-Nieto C., Cantero G., Pérez M., Te N., Noguera-Julián M., Guallar V., Valencia A. (2021). Protection against reinfection with D614-or G614-SARS-CoV-2 isolates in golden Syrian hamster. Emer. Microbes Infect..

[19] Selvaraj P., Lien C.Z., Liu S., Stauft C.B., Nunez I.A., Hernandez M., Nimako E., Ortega M.A., Starost M.F., Dennis J.U. (2021). SARS-CoV-2 infection induces protective immunity and limits transmission in Syrian hamsters. Life Sci. Alliance.

[20] Halfmann P.J., Kuroda M., Armbrust T., Accola M., Valdez R., Kowalski-Dobson T., Rehrauer W., Gordon A., Kawaoka Y. (2022). Long-term, infection-acquired immunity against the SARS-CoV-2 Delta variant in a hamster model. Cell Rep..

[21] Korber B., Fischer W.M., Gnanakaran S., Yoon H., Theiler J., Abfalterer W., Hengartner N., Giorgi E.E., Bhattacharya T., Foley B. (2020). Tracking changes in SARS-CoV-2 spike: Evidence that D614G increases infectivity of the COVID-19 virus. Cell.

[22] Plante J.A., Liu Y., Liu J., Xia H., Johnson B.A., Lokugamage K.G., Zhang X., Muruato A.E., Zou J., Fontes-Garfias C.R. (2021). Spike mutation D614G alters SARS-CoV-2 fitness. Nature.

[23] European Centre for Disease Prevention and Control (2021). https://www.ecdc.europa.eu/en/covid-19/variants-concern.

[24] Choudhary M.L., Vipat V., Jadhav S., Basu A., Cherian S., Abraham P., Potdar V.A. (2020). Development of in Vitro Transcribed RNA as Positive Control for Laboratory Diagnosis of SARS-CoV-2 in India. Indian J. Med. Res..

[25] Moreira L.V.L., de Souza Luna L.K., Barbosa G.R., Perosa A.H., Chaves A.P.C., Conte D.D., Carvalho J.M.A., Bellei N. (2021). Test on Stool Samples Improves the Diagnosis of Hospitalized Patients: Detection of SARS-CoV-2 genomic and subgenomic RNA. J. Infect..

[26] Shete A., Mohandas S., Jain R., Yadav P.D. (2021). A qualitative IgG ELISA for detection of SARS-CoV-2-specific antibodies in Syrian hamster serum samples. STAR Protoc..

[27] Deshpande G.R., Sapkal G.N., Tilekar B.N., Yadav P.D., Gurav Y., Gaikwad S., Kaushal H., Deshpande K.S., Kaduskar O., Sarkale P. (2020). Neutralizing Antibody Responses to SARS-CoV-2 in COVID-19 Patients. Indian J. Med. Res..

[28] Arora P., Kempf A., Nehlmeier I., Graichen L., Sidarovich A., Winkler M.S., Schulz S., Jack H.M., Stankov M.V., Behrens G. (2021). Delta variant (B. 1.617. 2) sublineages do not show increased neutralization resistance. Cell Mol. Immunol..

[29] Bager P., Wohlfahrt J., Rasmussen M., Albertsen M., Krause T.G. (2021). Hospitalisation associated with SARS-CoV-2 delta variant in Denmark. Lancet Infect. Dis..

[30] Twohig K.A., Nyberg T., Zaidi A., Thelwall S., Sinnathamby M.A., Aliabadi S., Seaman S.R., Harris R.J., Hope R., Lopez-Bernal J. (2022). Hospital admission and emergency care attendance risk for SARS-CoV-2 delta (B.1.617.2) compared with alpha (B.1.1.7) variants of concern: A cohort study. Lancet Infect. Dis..

[31] Polak S.B., Van Gool I.C., Cohen D., von der Thüsen J.H., van Paassen J. (2020). A systematic review of pathological findings in COVID-19: A pathophysiological timeline and possible mechanisms of disease progression. Mod. Pathol..

[32] Gruber A.D., Osterrieder N., Bertzbach L.D., Vladimirova D., Greuel S., Ihlow J., Horst D., Trimpert J., Dietert K. (2020). Standardization of reporting criteria for lung pathology in SARS-CoV-2–infected hamsters: What matters?. Am. J. Respir. Cell Mol..

[33] Wibmer C.K., Ayres F., Hermanus T., Madzivhandila M., Kgagudi P., Oosthuysen B., Lambson B.E., De Oliveira T., Vermeulen M., Van der Berg K. (2021). SARS-CoV-2 501Y. V2 escapes neutralization by South African COVID-19 donor plasma. Nat. Med..

[34] Harvey W.T., Carabelli A.M., Jackson B., Gupta R.K., Thomson E.C., Harrison E.M., Ludden C., Reeve R., Rambaut A., Peacock S.J. (2021). SARS-CoV-2 variants, spike mutations and immune escape. Nat. Rev. Microbiol..

[35] Yadav P.D., Sahay R.R., Sapkal G., Nyayanit D., Shete A.M., Deshpande G., Patil D.Y., Gupta N., Kumar S., Abraham P. (2021). Comparable neutralization of SARS-CoV-2 Delta AY. 1 and Delta in individuals sera vaccinated with BBV152. J. Travel Med..

[36] Rabaan A.A., Al-Ahmed S.H., Muhammad J., Khan A., Sule A.A., Tirupathi R., Mutair A.A., Alhumaid S., Al-Omari A., Dhawan M. (2021). Role of inflammatory cytokines in COVID-19 patients: A review on molecular mechanisms, immune functions, immunopathology and immunomodulatory drugs to counter cytokine storm. Vaccines.

[37] Merza M.Y., Hwaiz R.A., Hamad B.K., Mohammad K.A., Hama H.A., Karim A.Y. (2021). Analysis of cytokines in SARS-CoV-2 or COVID-19 patients in Erbil city, Kurdistan Region of Iraq. PLoS ONE.

[38] Santa Cruz A., Mendes-Frias A., Oliveira A.I., Dias L., Matos A.R., Carvalho A., Capela C., Pedrosa J., Castro A.G., Silvestre R. (2021). IL-6 is a biomarker for the development of fatal SARS-CoV-2 pneumonia. Front. Immunol..

[39] Francis M.E., Goncin U., Kroeker A., Swan C., Ralph R., Lu Y., Etzioni A.L., Falzarano D., Gerdts V., Machtaler S. (2021). SARS-CoV-2 infection in the Syrian hamster model causes inflammation as well as type I interferon dysregulation in both respiratory and non-respiratory tissues including the heart and kidney. PLos Pathog..

[40] Velazquez-Salinas L., Verdugo-Rodriguez A., Rodriguez L.L., Borca M.V. (2019). The role of interleukin 6 during viral infections. Front. Microbiol..

[41] Dan J.M., Mateus J., Kato Y., Hastie K.M., Yu E.D., Faliti C.E., Grifoni A., Ramirez S.I., Haupt S., Frazier A. (2021). Immunological memory to SARS-CoV-2 assessed for up to 8 months after infection. Science.

[42] Krammer F. (2020). SARS-CoV-2 vaccines in development. Nature.

[43] Muecksch F., Weisblum Y., Barnes C., Schmidt F., Schaefer-Babajew D., Lorenzi J., Flyak A., DeLaitsch A., Huey-Tubman K., Hou S. (2021). Affinity maturation of SARS-CoV-2 neutralizing antibodies confers potency, breadth, and resilience to viral escape mutations. Immunity.

[44] Zhang C., Guo Z., Li N., Cui H., Meng K., Liu L., Zhao L., Zhang S., Qin C., Liu J. (2021). Impact of Prior Infection on Severe Acute Respiratory Syndrome Coronavirus 2 Transmission in Syrian Hamsters. Front. Microbiol..

[45] Chandrashekar A., Liu J., Martinot A.J., McMahan K., Mercado N.B., Peter L., Tostanoski L.H., Yu J., Maliga Z., Nekorchuk M. (2020). SARS-CoV-2 infection protects against rechallenge in rhesus macaques. Science.

[46] Gao Q., Bao L., Mao H., Wang L., Xu K., Yang M., Li Y., Zhu L., Wang N., Lv Z. (2020). Development of an inactivated vaccine candidate for SARS-CoV-2. Science.

[47] Yu J., Tostanoski L.H., Peter L., Mercado N.B., McMahan K., Mahrokhian S.H., Nkolola J.P., Liu J., Li Z., Chandrashekar A. (2020). DNA vaccine protection against SARS-CoV-2 in rhesus macaques. Science.

[48] Cromer D., Juno J.A., Khoury D., Reynaldi A., Wheatley A.K., Kent S.J., Davenport M.P. (2021). Prospects for durable immune control of SARS-CoV-2 and prevention of reinfection. Nat. Rev. Immunol..

