# Supplementary material for: Protective Immunity of the Primary SARS-CoV-2 Infection Reduces Disease Severity Post Re-Infection with Delta Variants in Syrian Hamsters
Source: Viruses. 2022 Mar 13;14(3):596. doi: 10.3390/v14030596 (PMC8950956; doi:10.3390/v14030596)
Supplement: Supplementary file 1 [file viruses-14-00596-s001.zip › viruses-1604208-supplementary.pdf]

viruses

MDPI

Supplementary Materials

Protective Immunity of the Primary SARS-CoV-2 Infection Reduces Disease Severity Post Re-infection with Delta Variants in Syrian Hamsters

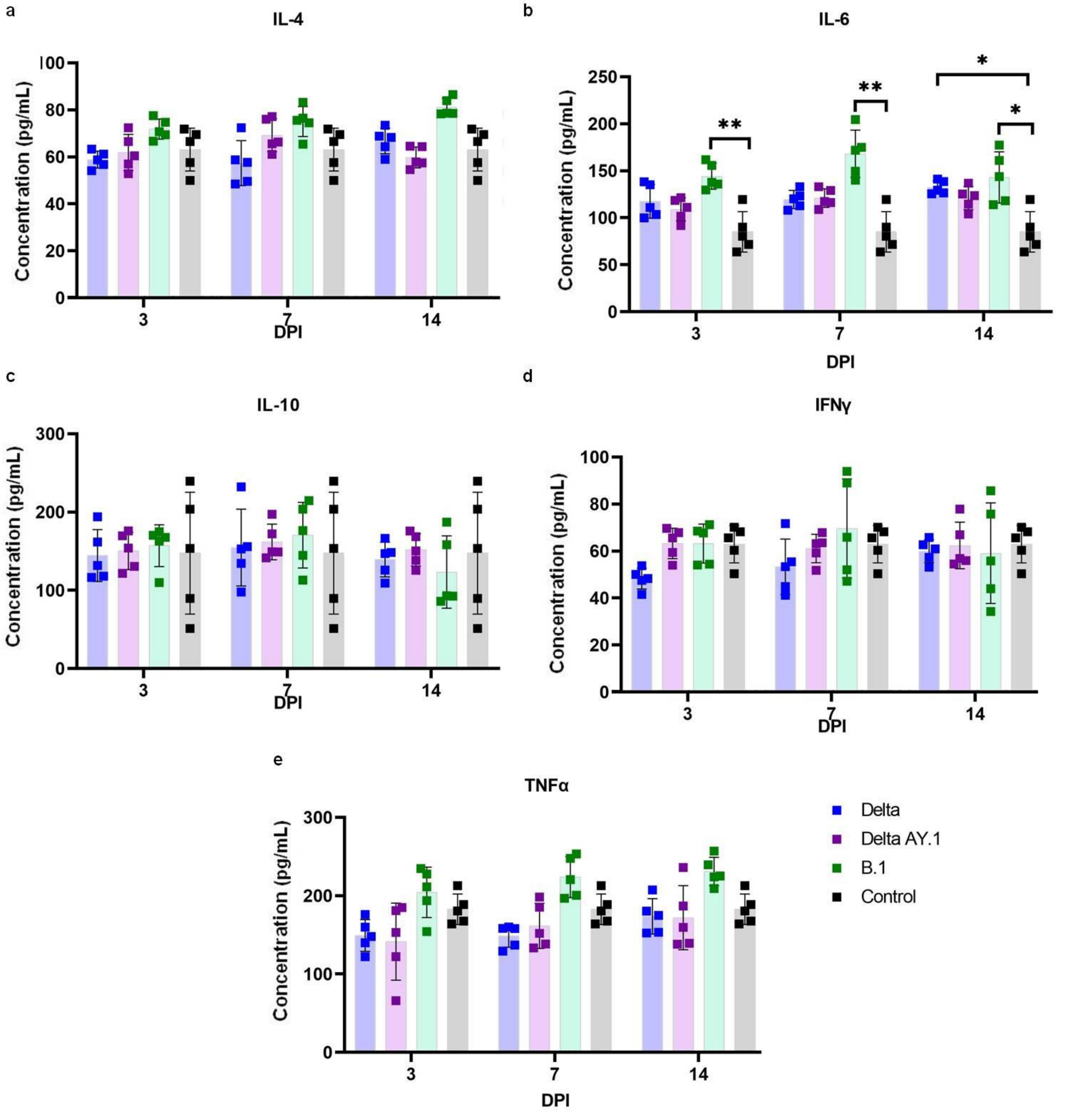

Figure S1. Serum cytokine levels in hamsters post SARS CoV-2 infection. Scatter plot depicting **a**) IL-4, **b**) IL-6 (p = 0.0037on 3 DPI, p = 0.0011 on 7 DPI and p = 0.0327 on 14 DPI, B.1 vs Control, p= 0.0277 on 14 DPI, Delta vs Control, Mann Whitney test, n = 5), **c**) IL-10, **d**) IFN-gamma **e**) TNF-alpha levels in serum of hamsters on 3, 7 and 14 DPI post infection. The bars represent the mean and the error bars depict the standard deviation. The p values < 0.05, < 0.001 and <0.0001 are represented as *, ** and *** respectively.

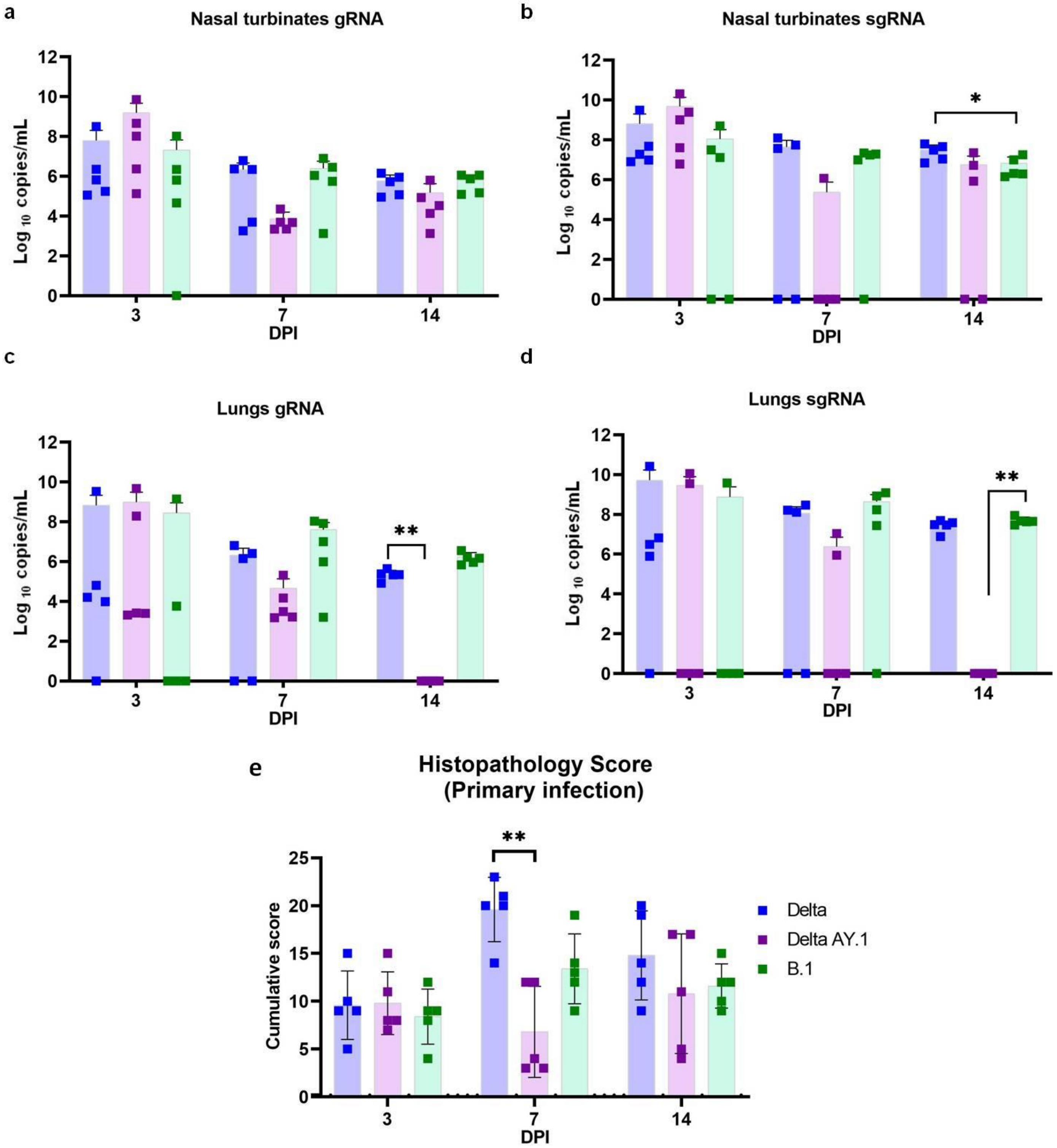

Figure S2. SARS-CoV-2 viral RNA load in organs of hamster's post infection. Scatter plot depicting viral gRNA load in hamsters **a**) nasal turbinates and **c**) lungs. Scatter plot depicting viral sgRNA

load in **b**) nasal turbinates (*p* = 0.0397, Delta vs B.1, Mann Whitney test, *n* = 5) and **d**) lungs (*p* = 0.0057, AY.1 vs B.1, Mann Whitney test, *n* = 5. **e**) Scatter plot depicting cumulative lung histopathology score in hamsters post primary infection on 3, 7 and 14 DPI (*p* = 0.0036, Delta vs Delta AY.1, Mann Whitney test, *n* = 5). The bars represent the mean and the error bars depict the standard deviation. The p values < 0.05 and < 0.001 are represented as * and **.

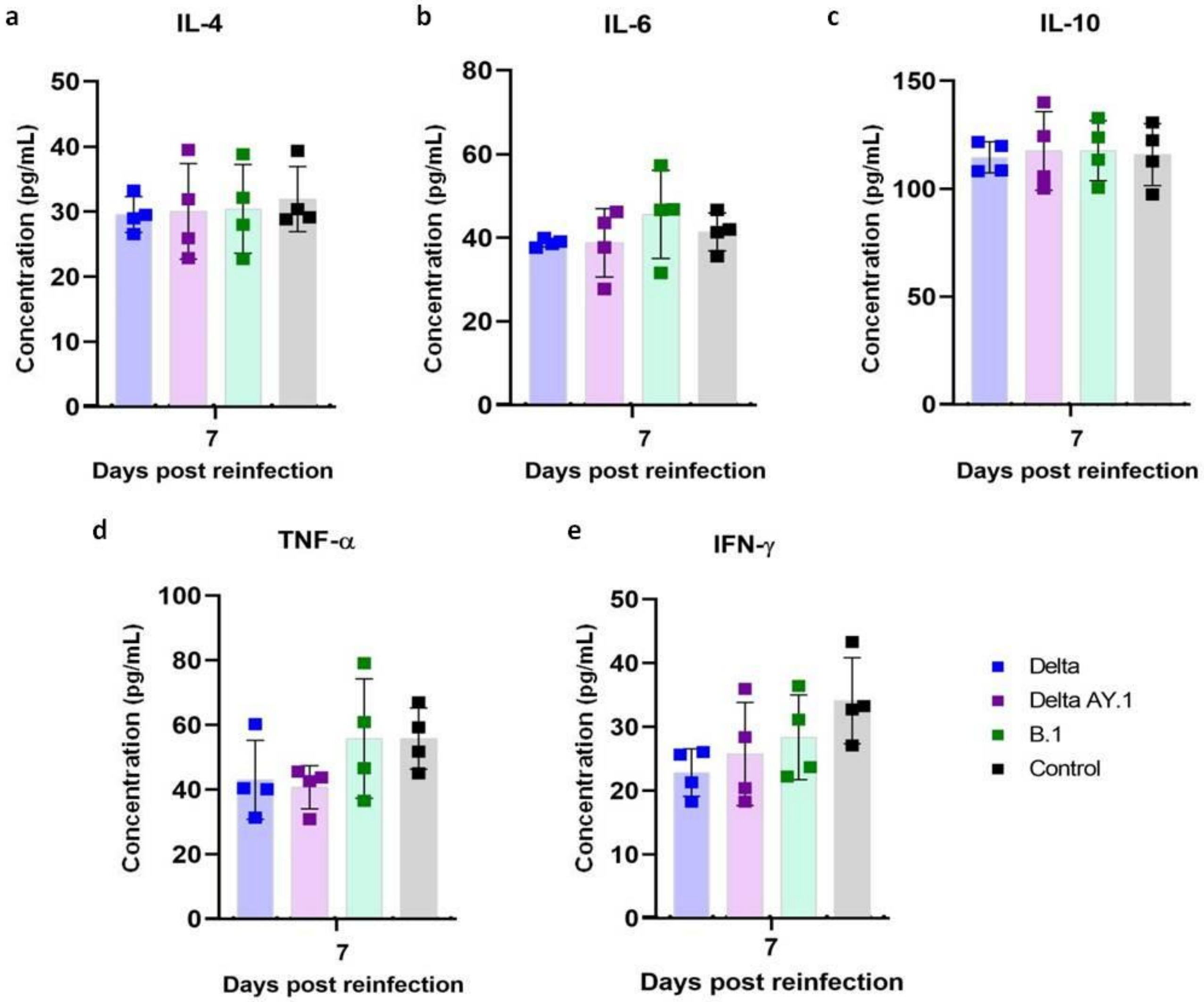

Figure S3. Serum cytokine levels in hamsters post SARS CoV-2 re-infection. Scatter plot depicting **a**) IL-4, **b**) IL-6 **c**) IL-10, **d**) TNF- α **e**) IFN- γ levels in serum of hamsters on 7-day post re-infection. The bars represent the mean and the error bars depict the standard deviation.

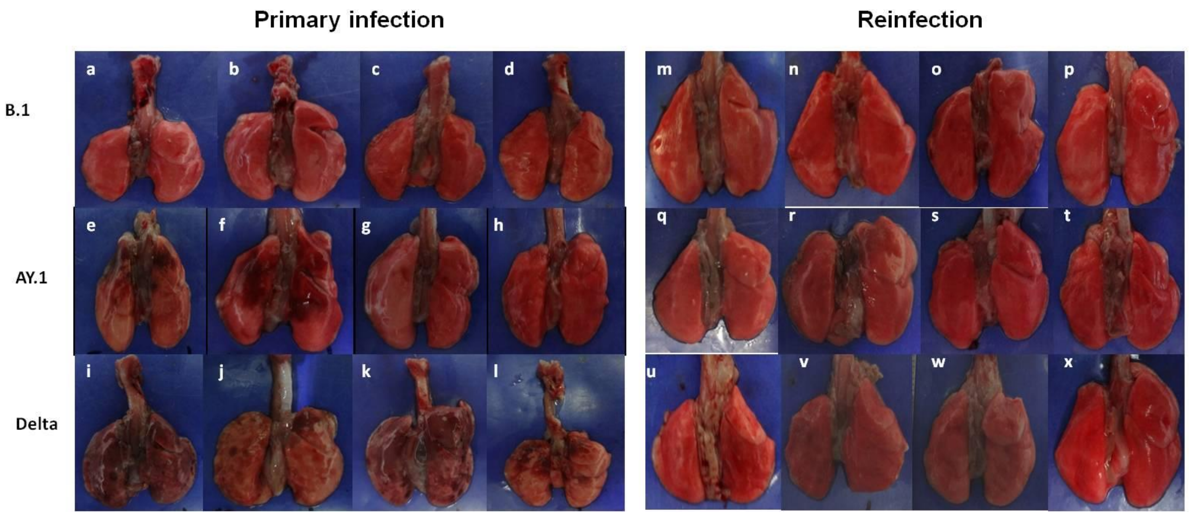

Figure S4. Pathological changes observed in lungs after primary infection and re-infection. Lungs showing (**a**-**d**) normal gross appearance following infection with B.1 variant, (**e**,**f**) haemorrhages in left and right lower lobes and (**g**,**h**) normal gross appearance in Delta AY.1 infected animals and (**i**-**l**) diffuse haemorrhages in all lung lobes in Delta infected animals. Lungs showing (**m**-**p**) showing normal gross appearance after B.1 re-infection, (**q**-**t**) showing normal gross appearance after Delta AY.1 re-infection and (**u**,**x**) showing normal gross appearance and (**v**,**w**) focal congestion after Delta infection.

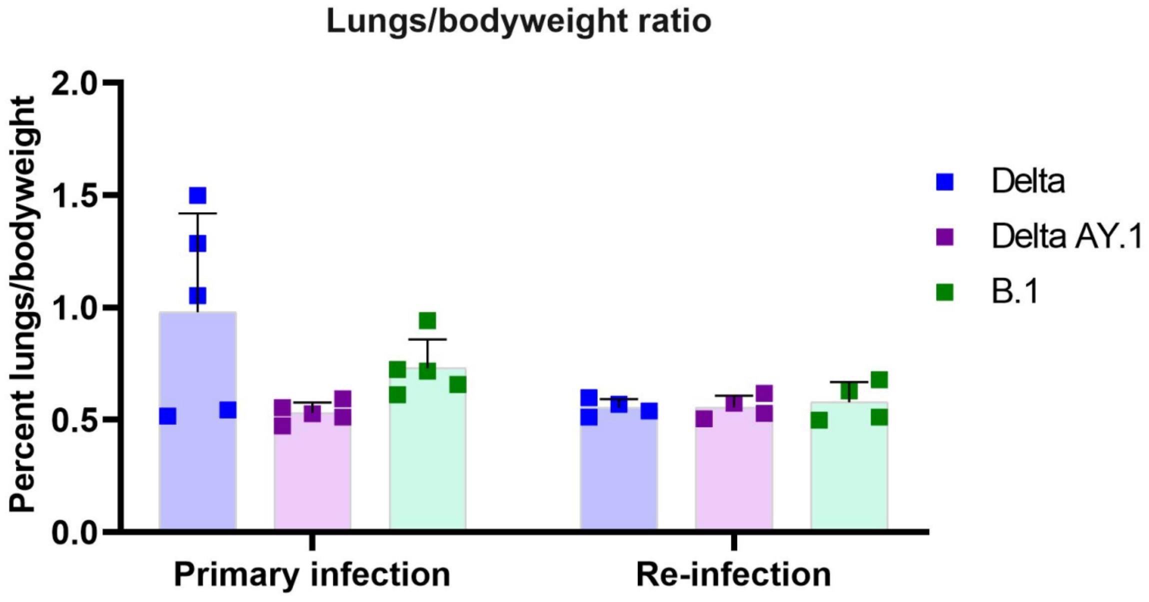

Figure S5. Lungs body weight ratio in hamsters after primary infection and re-infection.
